# Supplementary material for: From sequence to enzyme mechanism using multi-label machine learning
Source: BMC Bioinformatics. 2014 May 19;15:150. doi: 10.1186/1471-2105-15-150 (PMC4229970; doi:10.1186/1471-2105-15-150)
Supplement: Additional file 2 — Java code of ml2db. Additional file ml2db_code.tar.gz contains the Java source code to run the multi-label machine learning experiments and save the results to database. The code’s Javadoc is included. [file 1471-2105-15-150-S2.zip › additional file 2/ml2db/ml2db/doc/test/ml2db/alltests/package-summary.html]

test.ml2db.alltests


JavaScript is disabled on your browser.


- Overview
- Package
- Class
- Use
- Tree
- Deprecated
- Index
- Help

- Prev Package
- Next Package

- Frames
- No Frames

- All Classes

# Package test.ml2db.alltests

- Class Summary

  | Class | Description |
  | --- | --- |
  |  |  |
  | --- | --- |
  | AllActiveLearningTests | Class |
  | AllArffTests | Class |
  | AllDataTests | Data harness for arff file generation and learning tests. |
  | AllExternalPredictionsEvaluationTests | Class |
  | AllGraphVizTests | Class |
  | AllMulanArffTests | Class |
  | AllMulanLearningTests | Class |
  | AllMulanPredictionTests | Class |
  | AllMulanTests | Class |
  | AllPreliminaryTests | Class |
  | AllTests | All tests Note: RUN AllDataTests FIRST to generate the database tables, some arff files, some data etc. |
  | AllTrainTestsTests | Class |
  | DbManagerForTest |  |

- Overview
- Package
- Class
- Use
- Tree
- Deprecated
- Index
- Help

- Prev Package
- Next Package

- Frames
- No Frames

- All Classes
